# Supplementary material for: A New Mechanism of Carbon Metabolism and Acetic Acid Balance Regulated by CcpA
Source: Microorganisms. 2023 Sep 13;11(9):2303. doi: 10.3390/microorganisms11092303 (PMC10535407; doi:10.3390/microorganisms11092303)
Supplement: Supplementary file 1 [file microorganisms-11-02303-s001.zip › microorganisms-2566157-supplementary.pdf]

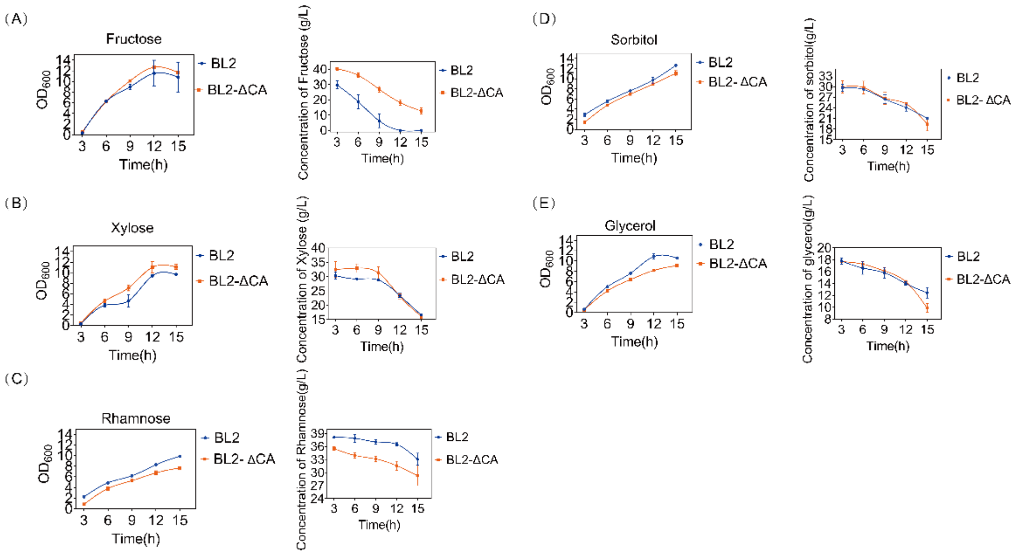

Figure S1. Comparison of consumption of different carbon sources by CcpA defective strains. Figure S1A: Fructose consumption and OD_{600} of *Bacillus licheniformis* and *Bacillus licheniformis CA* in medium with fructose. Figure S1B: Xylose consumption and OD_{600} of *Bacillus licheniformis* and *Bacillus licheniformis CA* in medium with xylose. Figure S1C: Rhamnose consumption and OD_{600} of *Bacillus licheniformis* and *Bacillus licheniformis CA* in medium with rhamnose. Figure S1D: Sorbitol consumption and OD_{600} of *Bacillus licheniformis* and *Bacillus licheniformis CA* in medium with sorbitol. Figure S1E: Glycerol consumption and OD_{600} of *Bacillus licheniformis* and *Bacillus licheniformis CA* in medium with glycerol.
